# Modelling compressive loading in large‐bodied mammals reveals distinct biomechanical strategies in proboscidean humeri

**DOI:** 10.1111/joa.70234

**Published:** 2026-09-07

**Authors:** Nicolas Ferdinand Wagner, Narimane Chatar, Jesse James Hennekam

**Affiliations:** ^1^ Maastricht Science Programme, Faculty of Science and Engineering Maastricht University Maastricht the Netherlands; ^2^ Comparative Zoology Institut für Biologie, Humboldt‐Universität zu Berlin Berlin Germany; ^3^ Department of Integrative Biology University of California Berkeley Berkeley California USA; ^4^ Departamento de Ecología y Geología, Facultad de Ciencias Universidad de Málaga Málaga Spain; ^5^ Understanding Evolution Naturalis Biodiversity Center Leiden the Netherlands

**Keywords:** axial compression, elephants, FEA, finite element analysis, functional morphology, graviportal, *Loxodonta*, *Mammuthus*

## Abstract

Proboscideans exhibit limb morphologies specialised for supporting extreme body masses under predominantly compressive loading. These graviportal adaptations include repositioning of the limb bones closer to the trunk, resulting in a columnar stance. Among proboscideans, two humeral morphotypes have been proposed (slender and robust), yet their functional significance under compressive loading remains poorly understood. Finite element analysis (FEA) is applied to evaluate biomechanical performances of proboscidean humeri subject to axial compression, simulating conditions associated with a columnar stance and deviations from it. Our results reveal clear differences between morphotypes, with slender humeri performing worse than the robust morphotype and functioning optimally under near‐axial loading. Robust humeri consistently exhibit lower peak stress under both neutral and off‐axis loading and show considerable resistance to mediolateral stresses. Across all taxa, stress magnitudes generally increase when deviating from a neutral orientation, especially during posterior loading orientations, highlighting the mechanical importance of maintaining columnar limb posture. These findings support the hypothesis that robust morphologies are better adapted to resist multidirectional stresses, while slender morphologies favour energetic and locomotor efficiency. This study demonstrates the applicability of FEA in assessing compressive performance in postcranial elements and provides a novel framework for investigating biomechanical adaptations in graviportal taxa.

## Graphical abstract

Graviportal proboscideans differ in limb morphology, adopting slender or more robust long bone morphotypes. Finite element analyses (FEA) on extant and extinct taxa reveal functional differences between morphotypes, with robust humeri exhibiting lower peak stress under neutral and off‐axis loading. This study demonstrates the applicability of FEA in assessing compressive performance in postcranial elements and provides a novel framework for investigating biomechanical adaptations in graviportal taxa.

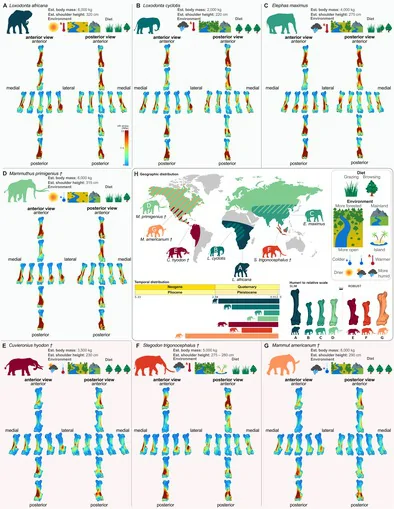

## 1 INTRODUCTION

The order Proboscidea (i.e. elephants, mammoths and mastodons) encompasses some of the largest terrestrial animals, including species that could reach shoulder heights of over 5 m and weigh over 20 tons (Larramendi, 2016). They are often regarded as the classic example of ‘graviportality’ (Alexander & Pond, 1992; Gregory, 1912), a term introduced specifically to characterise animals exhibiting a body mass of several thousand kilograms, the inability to gallop, robust bones and a columnar stance (Alexander & Pond, 1992; Bader et al., 2024; Christiansen, 2007; Gambaryan, 1974; Herridge, 2010; Kokshenev & Christiansen, 2010). This columnar limb orientation is a solution adapted by many heavy terrestrial animals across the fossil record (Carrano, 1998; Christiansen, 2007; Coombs, 1978; Sander et al., 2011), as it favours axial compression over more complex bending and torsional moments (Christiansen, 2007; Kokshenev & Christiansen, 2010).

Two distinct limb morphotypes are proposed to differentiate among proboscidean taxa (Bader et al., 2024): one ‘slim’ morphotype, featuring relatively long, slender limbs with thin diaphyses and narrow epiphyses and one ‘robust’ morphotype, displaying thicker diaphyses and wider epiphyses for a given limb length. Only the slim morphotype is represented among extant, relatively lightweight proboscideans today, whereas both morphotypes were present in heavier species in the fossil record (Bader et al., 2024). Interestingly, many taxa can be fully categorised as slim or robust (such as *Elephas maximus* and *Mammut americanum*, respectively), while other species exhibit an intermediate configuration (Bader et al., 2024). These species do not appear to represent a transitional morphology between the two morphotypes; rather, they indicate that some elements of a species are categorised as robust, while other elements are categorised as slim. This is observed in *Stegodon trigonocephalus*, for example, where the humerus is robust, whereas the zeugopod elements exhibit a slimmer morphology (Bader et al., 2024).

The slender morphotype is expected to better withstand axial loading associated with the columnar limb orientation. In contrast, the robust morphotype would increase resistance to multidirectional bending stresses and is associated with greater muscular forces and a less columnar stance, implying an increased reliance on muscle‐driven stabilisation (Bader et al., 2024). Observational evidence shows that extant elephants exhibit a relatively ‘narrow‐gauge’ gait, with their limbs angled inward (medially), although they can place their feet in various orientations when stationary (Florides & Christodoulides, 2021). This preferred stance may explain the distinctly larger diaphyses in the lateromedial (rather than the anteroposterior) diameters, suggesting increased structural strength in these positions (Christiansen, 2007).

**FIGURE 1:**
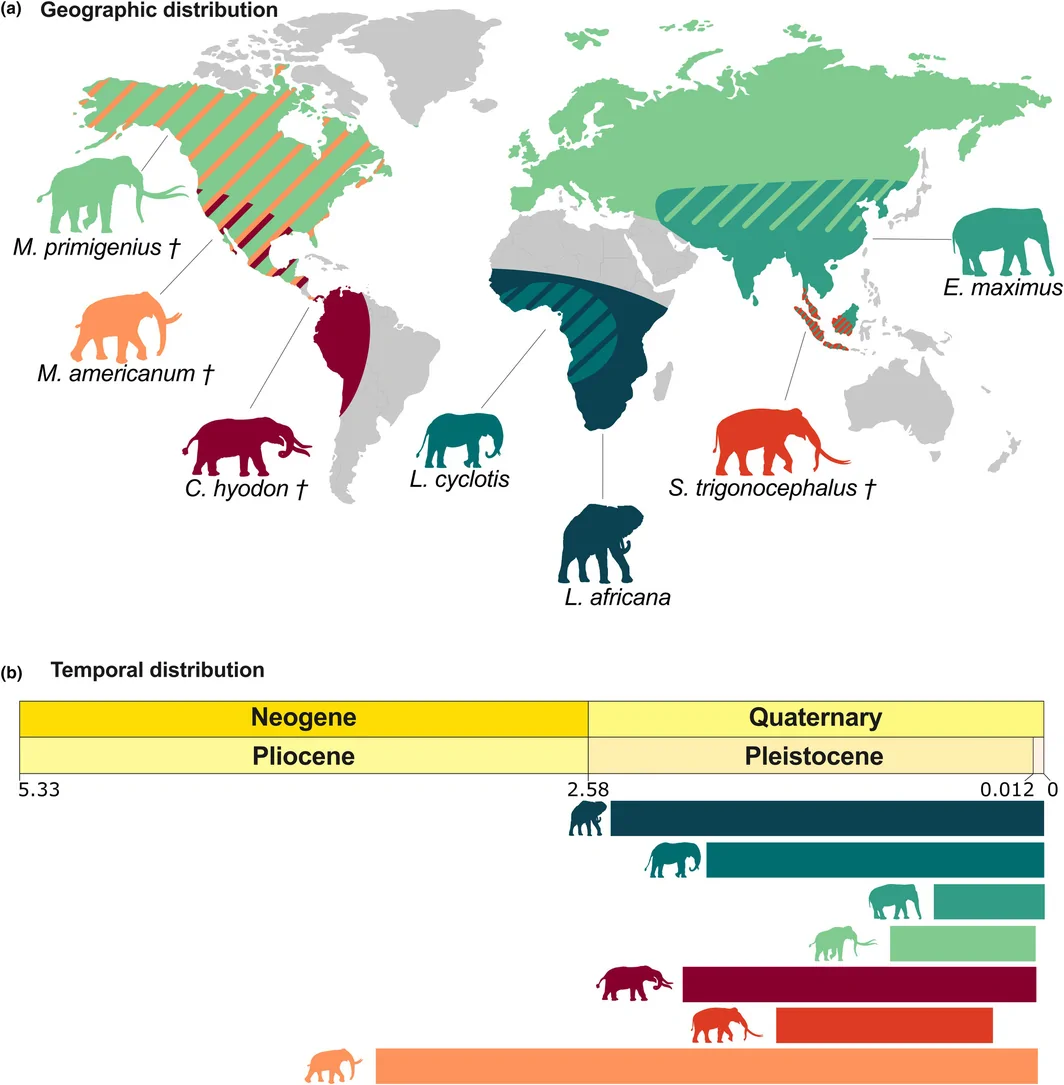
(a) Map showing the geographic distributions and (b) the temporal occurrences of the species *Loxodonta africana*, *Loxodonta cyclotis*, *Elephas maximus*, *Mammuthus primigenius*, *Cuvieronius hyodon*, *Stegodon trigonocephalus* and *Mammut americanum*. Species marked with a dagger (†) are extinct.

Proboscideans occupied a broad range of environments, from dense tropical rainforests and wooded wetland environments to open savannahs and the cold, open mammoth steppe (Figure 1; Budd et al., 2020; Kahlke, 2015; Krishnan et al., 2019; Newsom & Mihlbachler, 2006; Puspaningrum et al., 2020; Roocroft & Oosterhuis, 2000). The earliest proboscideans are known from the Palaeocene of Africa (Gheerbrant, 2009). However, these early‐diverging lineages were not uniformly large‐bodied or graviportal. The earliest species were considerably smaller than modern elephants, and increases in body mass were not equally pronounced across all lineages (Larramendi, 2016). Sustaining their large body sizes requires high absolute food intake, which in turn promotes near‐continuous foraging and extensive daily movement, often supplemented by seasonal migrations (Hodgson et al., 2008; Kokshenev & Christiansen, 2010; McKay, 1973; Owen‐Smith, 1988; Price, 2022; Vancuylenberg, 1977; Wooller et al., 2021). Variation in landscape structure and heterogeneity is therefore suspected to influence both migratory distances and the mechanical demands placed on the locomotor system (Kokshenev & Christiansen, 2010), necessitating locomotor strategies capable of accommodating diverse substrates and inclines. Forelimb morphology has been suggested as a good indicator of terrestrial locomotor ecology, as they support a substantial portion of the body's weight and are critical for stability and shock absorption on ground impact (Hooper & Schulz, 2020; MacLaren & Nauwelaerts, 2016). Variation in forelimb morphology of proboscideans is expected to reflect adaptations to their size, locomotion and stance (Bader et al., 2023), which in turn can reflect responses to specific habitats.

Weight distribution within proboscideans is significantly impacted by sexual dimorphism, with males being approximately 15%–23% heavier than females, a pattern driven by factors, such as tusk size and mass (Larramendi, 2016; Larramendi & Palombo, 2015). The forelimbs of elephants carry more weight than the hindlimbs (Bader et al., 2023, 2024, 2025; Ren et al., 2010), resulting in a roughly 60/40 weight distribution (Genin et al., 2010; Hooper & Schulz, 2020; Ren et al., 2010). Across the skeleton of heavy terrestrial mammals, the forelimb shows stronger and more consistent adaptations to weight bearing than the hindlimb (Bader et al., 2023, 2024; Mallet et al., 2020). Thickened cortical bone along the medial shaft and the deltoid tuberosity has been found for *Loxodonta africana* and *E. maximus*, as well as trabecular bone oriented from the thick trabecular cortex to the lateral supracondylar crest (Bader et al., 2025). This thicker cortex allows for better resistance to axial compression, and is therefore hypothesised to correspond to areas of highest stress under loading (Bader et al., 2025). These findings highlight the need to evaluate whether proboscideans relied on different mechanical strategies for weight support, and to determine whether the slender and robust morphotypes exhibit measurable differences in structural performance under compressive loading regimes.

Extinct graviportal proboscideans show more diversification in body mass than extant taxa, ranging from 1‐m‐tall dwarfed taxa on islands (Larramendi, 2016; Larramendi & Palombo, 2015) to the impressive body size of *Deinotherium giganteum* or *Mammut borsoni* (Larramendi, 2016). This variation in body sizes is often accompanied by distinct body proportions, leading to notable differences in the relationship between body mass and bone dimensions (Bader et al., 2024; Larramendi, 2016). This variation in body size and limb bone shape among proboscideans will influence the structural performance of the limbs during compressional loading. The morphological variation among proboscideans arises from a combination of factors, including the acquisition of columnar limb posture and the biomechanical demands of gigantism, both of which drive consistent changes, such as straighter shafts and reoriented articular surfaces (Bader et al., 2024). This variation among species is not solely explained by differences in body size and mass (Bader et al., 2023, 2024). Instead, it reflects the evolution of two distinct morphotypes in proboscideans, representing alternative adaptations for supporting high body weight, each of which tends to remain conserved once established within a lineage. Within morphotypes, morphological variation persists. Extant proboscideans display only the slender morphotype; however, within extant elephants, the Asian elephant exhibits unusually robust limb bones relative to their mass, presumably in response to habitat‐related functional demands (Bader et al., 2023).

Proboscidean limbs represent one of the most extreme expressions of graviportal adaptation (Alexander & Pond, 1992; Bader et al., 2024) and provide a relatively straightforward framework for compressional loading, making them ideal candidates for functional studies centred on compressive loading (Marcé‐Nogué, 2022). Finite element analysis (FEA) is an effective way to simulate this compressive loading, as it can simulate the stresses and strains experienced under the massive body weights characteristic of these animals (Marcé‐Nogué, 2022). While FEA has been applied in diverse studies of cranial biomechanics across the animal kingdom (Brunt et al., 2016; Chatar et al., 2022; Habegger et al., 2020; Hennekam et al., 2023; Lautenschlager, 2022; Marcé‐Nogué et al., 2017; O'Higgins et al., 2019; Rowe & Rayfield, 2025; Soons et al., 2012; Tseng & Terhune, 2024), its use for analysing limb bones under compressive forces in palaeontology is still emerging (Silva Junior et al., 2025).

The limited understanding of how limb bones withstand and distribute compressive forces under high body mass constitutes a gap in our understanding of skeletal biomechanics. This study represents a novel application of FEA to assess the compressive performance of proboscidean humeri at different loading orientations. We predict that robust humeri outperform the slim ones, and that performance will be highest when the bone is either straight or slightly medially inclined, as this orientation reflects the natural stance observed in extant proboscideans. Conversely, greater deviation from this optimal alignment is expected to reduce performance. Additionally, regional microanatomical fortifications of the humerus are expected to correspond to areas experiencing higher stresses. If confirmed, those results would demonstrate the potential of this approach to yield meaningful inferences for stance and loading distributions in proboscideans. More broadly, this framework could provide insights into the structural and functional adaptations that enable these taxa to support and move such large masses, potentially making it applicable to other graviportal taxa, such as sauropod dinosaurs or Paraceratheriidae, the largest land mammals ever to roam the earth.

## 2 METHODS

### 2.1 Institutional abbreviations

MNHN—Paris: Muséum National d'Histoire Naturelle—Paris, France; RMCA: Royal Museum for Central Africa, Belgium; ML HL H: Collection Dick Mol, Mammoth Laboratory, Historyland, Hellevoetsluis, the Netherlands; IMNH: Idaho Museum of Natural History, United States of America.

### 2.2 Sample and scanning

We analysed nine loading conditions for a total of seven specimens representing seven different species across six genera (Table 1). *Loxodonta cyclotis*, *Mammuthus primigenius, Mammut americanum* and *S. trigonocephalus* were scanned with the Artec Leo (MorphoSource ID: 000853405). The remaining three specimens were acquired from the MorphoSource repository (Boyer et al., 2017), including *L. africana* and *Cuvieronius hyodon* (Artec Eva: MorphoSource ID: 000632460 and 000631088, respectively) and *E. maximus* (Faro Edge Arm: MorphoSource ID: 000168768). The dataset includes both extant and extinct taxa, representing four species characterised by the ‘slim’ morphotype and three by the ‘robust’ morphotype as per Bader et al. (2024) (Table 1). The exact age, shoulder height and body mass of each specimen were unknown, and expected adult specimens were chosen based on completed epiphyseal fusion. Estimates of body mass and shoulder height were obtained from Larramendi (2016) using values for Grade I (average‐sized, at the 90th percentile) male specimens.

**TABLE 1:** Specimen ID for each specimen, including morphotype (as assigned by Bader et al. (2024)), estimated body mass and shoulder height (as calculated by Larramendi (2016)), scanning technique and number of tetrahedra for the finite element (FE) models.

Species	Specimen ID	Institution	Morphotype	Est. BM in kg	Est. shoulder height in cm	Scanning technique	Number of tetrahedra
*Loxodonta africana*	mnhn:zm:AC‐1907‐49	MNHN—Paris	SLIM	6000	320	Artec Eva	2.2 × 10^6^
*Loxodonta cyclotis*	BE RMCA Vert.M.17772	RMCA	SLIM	2000	220	Artec Leo	2.7 × 10^6^
*Elephas maximus*	imnh:1486	IMNH	SLIM	4000	275	Faro Edge Arm	2.1 × 10^6^
*Mammuthus primigenius†*	ML 202503	ML HL H	SLIM	6000	315	Artec Leo	2.7 × 10^6^
*Cuvieronius hyodon†*	mad:f:TAR2514	MNHN—Paris	ROBUST	3500	230	Artec Eva	2.6 × 10^6^
*Stegodon trigonocephalus†*	ML 202502	ML HL H	ROBUST	5000	275	Artec Leo	2.6 × 10^6^
*Mammut americanum†*	ML 202501	ML HL H	ROBUST	8000	290	Artec Leo	2.5 × 10^6^

*Note*: Species marked with a asterisk (†) are extinct.

### 2.3 Preparation of the 3D data

**FIGURE 2:**
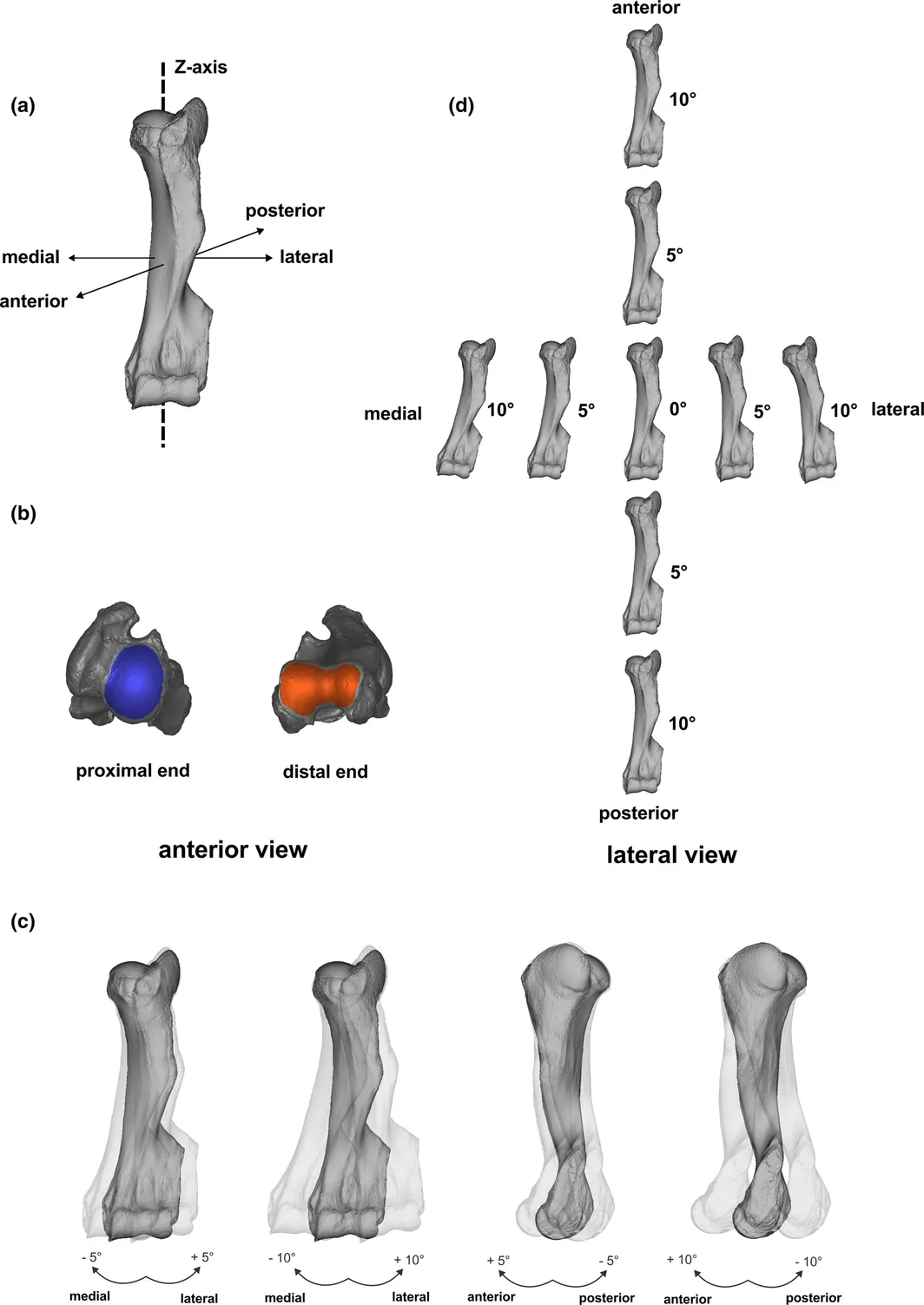
Humerus of *Elephas maximus* portraying the (a) orientation of the humeri (*Z*‐axis defines the loading direction), (b) the proximal and distal surfaces used for loading and fixating the models for finite element analysis (FEA), (c) the tilting procedure used for each specimen and (d) the resulting figure showing 0°, 5° and 10° tilts in each direction.

To enable FEAs, the meshes were made watertight, free of non‐manifold edges and self‐intersecting faces (Chatar et al., 2023) using Artec Studio 18 (Version 18; Artec 3D, 2023). All humeri were scaled to the length of the *E. maximus* humerus (766 mm) to ensure all bones experienced comparable loading conditions and thereby mitigate size bias. Length, rather than volume, was used because we wanted to test the impact of robustness (the ability of a structure to withstand a load) under similar loading conditions. Scaling was done in Blender (Version 4.3; Blender Online Community, 2024) using the ‘*Scale to Length*’ tool. The length of the humerus of *E. maximus* was defined as the distance between the centre of the *Trochlea humeri* and the top of the *Caput humeri* (Figure S1). In Artec Studio 18 (Version 18; Artec 3D, 2023), all models' *XYZ* coordinate systems were standardised. The alignment with the *Z*‐axis was performed as follows: with the humerus in dorsal (top) view (in orthographic, not parallel view), the centre of the head of the humerus (*Caput humeri*) was aligned with the coordinate origin (0,0,0). Then, using the ‘*Rotate*’ tool, the centre of the trochlea (*Trochlea humerus*) was aligned to 0,0,*Z* (see Figure 2).

The functional advantages of different limb orientations were tested by tilting each humerus in various directions: the distal end of the bone was tilted into the anterior, posterior, medial and lateral directions (reflecting protraction, retraction, adduction and abduction, respectively; Figure 2c) by both 5° and 10°, resulting in a total of 9 simulations per specimen and 63 in total (Figure 2d). Following those rotations, the same framework was employed for all seven specimens.

### 2.4 FEA protocol

The biomechanical performance will be assessed using FEA in the simulation software ‘Metafor’ (Version: v3541) developed at the University of Liège (Chatar et al., 2023) that has since been used in multiple studies (Chatar et al., 2024; de León‐Muñoz et al., 2025; Della Giustina et al., 2026; Silva Junior et al., 2025). FEA is a computational modelling technique widely used to analyse how physical structures respond to various external forces (Chatar et al., 2023; Grosse et al., 2007; Hennekam et al., 2023; Parashar & Sharma, 2016; Rayfield, 2007; Thomason, 1991; Tseng, 2009). It is particularly valuable for studying the biomechanical properties of fossilised bones and other skeletal elements in palaeontology. By creating 3D models from laser scans, the skeletal response of extinct organisms to stresses such as biting, walking or carrying weight can be simulated (Bright, 2014). Although these models are not literal representations of reality, they allow for inferences about the organisms' behaviour, physiology and evolutionary adaptations (Anderson et al., 2011).

Each surface mesh was decimated to ~250,000 faces, resulting in roughly 2.7 million tetrahedra, producing a very detailed model (preserving all anatomical details of the bones) (Tseng et al., 2011; Tseng & Flynn, 2015). This resolution is also substantially higher than in many recently published FEA studies modelling more complex structures (e.g. 300,000 tetrahedra for dinosaur skulls (Lautenschlager et al., 2025), 1 million tetrahedra for carnivoran skulls (Figueirido et al., 2025), 60,000–420,000 tetrahedra for marine reptile vertebrae (Wintrich et al., 2019), or similar bone, such as 75,000–92,000 elements for marine reptiles' humeri and femora (Krahl et al., 2022)).

In Metafor, this meshing step is done by silently calling Gmsh (Geuzaine & Remacle, 2009), a finite element mesh generator that includes integrated pre‐ and post‐processing functionalities. Forces of 23,544 N were chosen, as this constitutes 60% of *E. maximus*' estimated body mass (as calculated by Larramendi (2016)), representing the elephant resting its body weight on one limb (given the 60/40 weight distribution measured in Genin et al. (2010)). Forces were applied along the negative *Z*‐axis using the ‘Directional Traction Model’ in Metafor (Version: v3541; Chatar et al., 2023), as this approach distributes forces across the model nodes and scales them to match the specified target force. The proximal surface of each specimen was designated for force application, while the distal surface was fixed by constraining all degrees of freedom (Figure 2). Those regions were selected using the ‘lasso’ tool in Artec Studio 18, selecting the distal and proximal surfaces of each humerus and exporting those as STL files. The model was considered to be homogeneous cortical bone, with Young's modulus of 18 GPa and Poisson's ratio of 0.3 as defined by (Chatar et al., 2022; Erickson et al., 2002; Tseng et al., 2011).

For each test, we extracted three values: (1) the von Mises stress, which is an indicator of structural strength and a predictor of failure in biological structures. Therefore, a model exhibiting lower von Mises stress under identical loading conditions performs better than one with higher stress levels. (2) the displacement in the *Z*‐axis, which was used to quantify dorsoventral contraction during compression. Because all models were scaled to the same length prior to analysis, *Z*‐axis displacement is directly proportional to engineering (Cauchy) strain (defined as the change in length divided by the original length). Thus, displacement provides an equivalent measure for comparing compressive deformation among models. A lower displacement value suggests less compression and, therefore, better resistance to compressive loads. (3) The adjusted internal energy (as seen in Chatar et al., 2024; Della Giustina et al., 2026), which describes the amount of energy expended by the external forces to deform a structure and transfer loads through it. A high adjusted internal energy indicates that more energy is retained within the model, suggesting poor energy transfer. Conversely, a low adjusted internal energy signifies less energy storage within the structure and reduced deformation, demonstrating better performance under compressive load.

## 3 RESULTS

**FIGURE 3:**
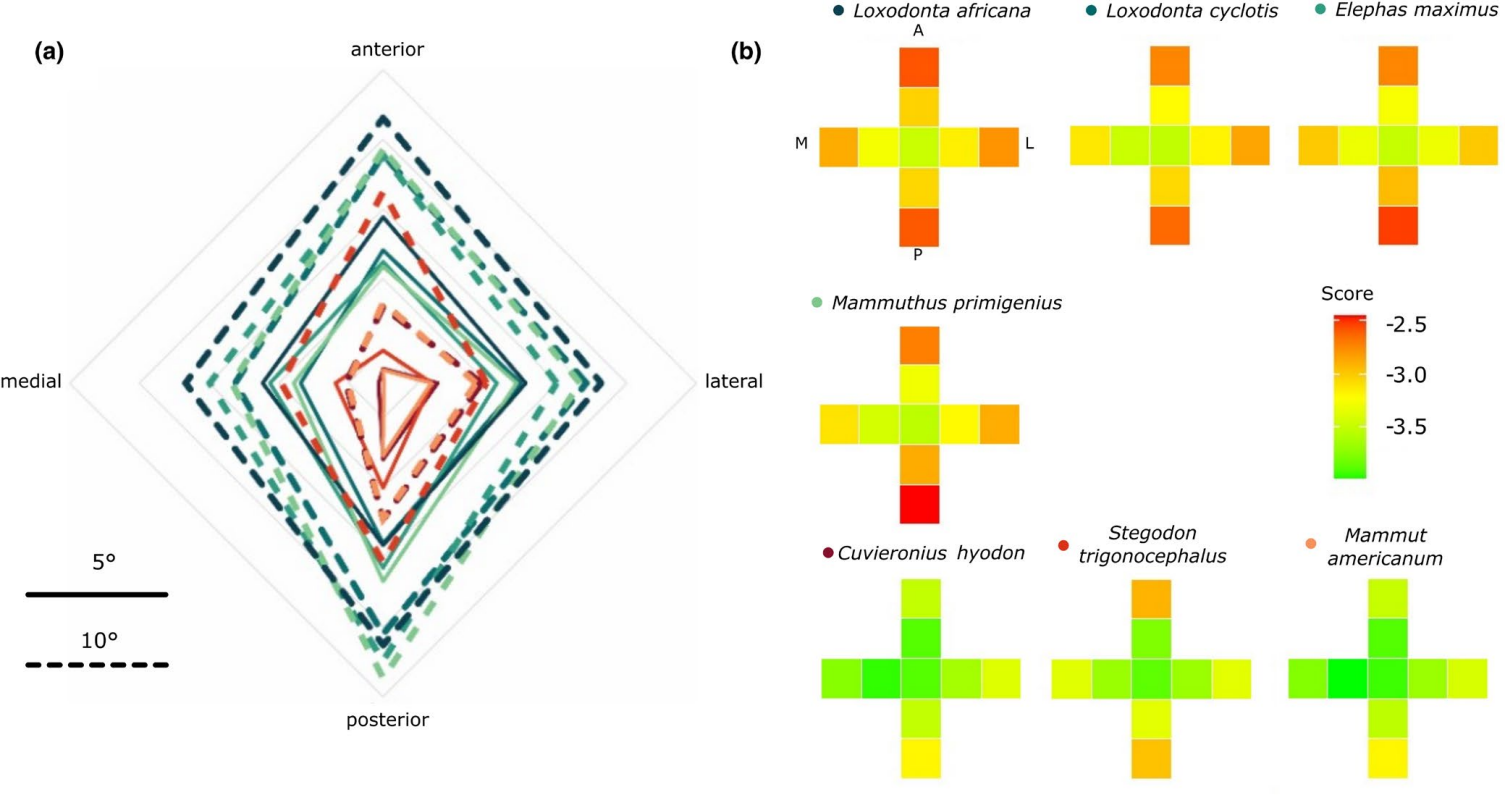
(a) Radar chart of the negative log‐transformed values for the adjusted internal energy for each proboscidean species in every rotational direction. Solid lines indicate a 5° tilt and dashed lines indicate 10° tilt in the anterior, medial, posterior and lateral direction. (b) Crosses showing logged adjusted internal energy values for each species for every tilting orientation. Red indicates higher values and thus worse mechanical performance, whereas green indicates a lower internal energy and indicative for better resistance to axial compression. The central square in each cross represents 0° of tilt, and the adjacent squares indicate 5° and 10°, respectively. A, anterior tilt; L, lateral tilt; M, medial tilt; P, posterior tilt. See Tables S1 and S2 for scaled internal energy (mJ) values and volume adjusted internal energy (mJ/mm^3^) values.

Across models, stress distributions varied with bone morphology and loading orientation. In all cases, slim humeri exhibited higher stress magnitudes than robust humeri in similar orientations (Figure 3; Figures S2 and S3). In general, orientations involving anterior and posterior tilts consistently produced higher stresses, whereas neutral loading and moderate medial or lateral tilts (5°) typically resulted in lower and more localised stress distributions. More extensive tilting (10°) consistently increased humeral loading compared with moderate tilting (5°). Across all species, stress distributions concentrated more distally, along the medial and posterior side of the shaft, as well as along the musculospiral groove (Figure S2).

### 3.1 Slim humeri

**FIGURE 4:**
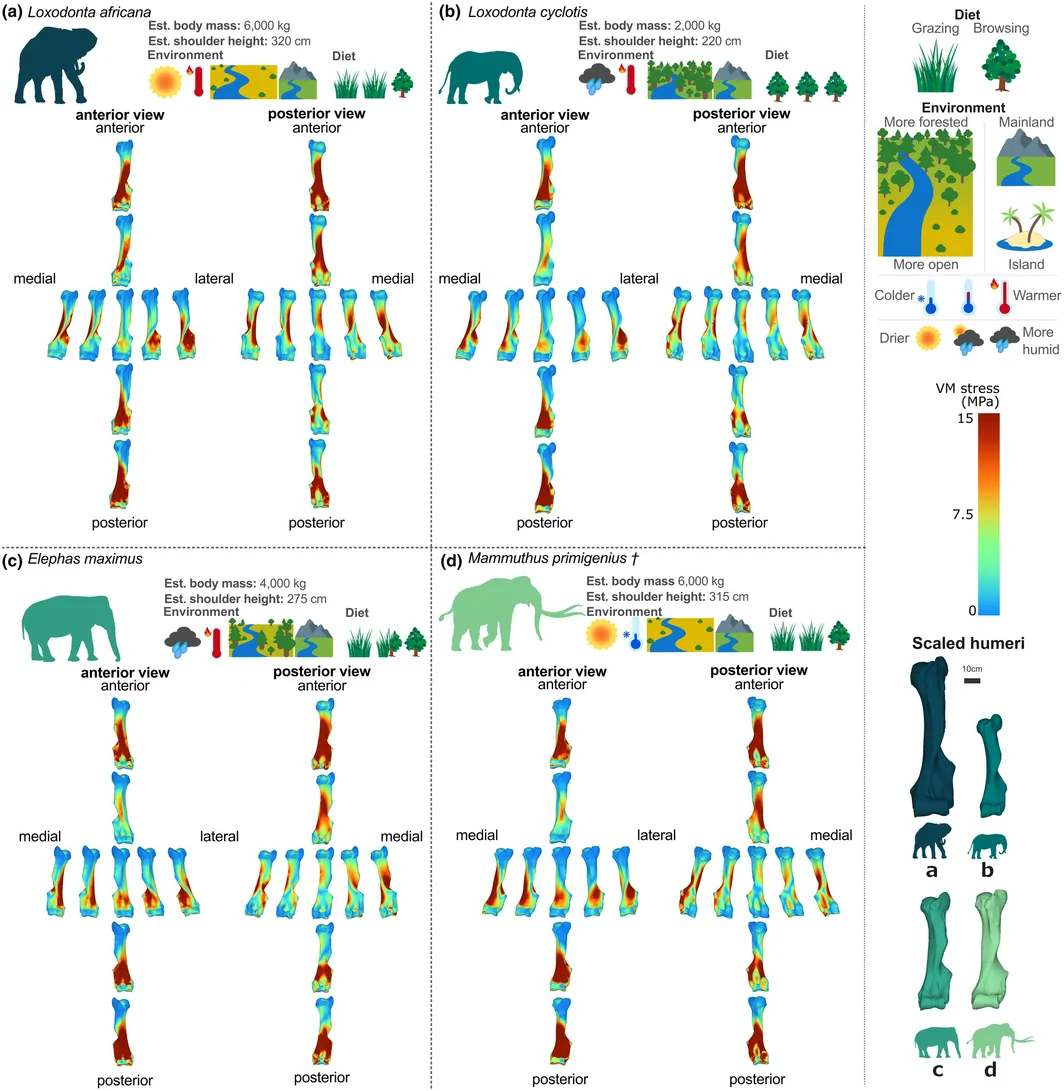
Results of the finite element (FE) analyses for the species exhibiting the slim morphotype (a, *Loxodonta africana*; b, *Loxodonta cyclotis*; c, *Elephas maximus*; d, *Mammuthus primigenius*). Species marked with a dagger (†) are extinct. Each panel highlights the diet and environment, as well as the relative size of the humeri to each other. Estimates of body mass and shoulder height were obtained from Larramendi (2016), based on Grade I (‘average‐sized’) male specimens, when possible.

Overall, slim humeri showed significant sensitivity to changes in loading orientation, with anterior and posterior tilts consistently producing the highest stresses, while neutral orientations experienced the lowest stress. Among slim taxa, the humerus of *L. africana* experienced the highest stresses, whereas *L. cyclotis* showed the least amount of stress. Humeri in the neutral orientation showed less stress than in off‐axis loading for all species, with comparatively minor loading in the musculospiral groove and the medial portion of the shaft (Figure 4). The 10° posterior and anterior orientations show the highest amounts of stress, as is exhibited by their adjusted internal energy (Figure 3).

#### 3.1.1 Loxodonta cyclotis

In *L. cyclotis*, neutral loading and a 5° medial tilt performed best, showing relatively low and localised stresses. In contrast, a 10° medial tilt resulted in notably higher stress, primarily concentrated laterally along the shaft (Figure 4). The 5° anterior tilt performed well when viewed anteriorly, whereas the posterior view showed substantial stress concentrations along the medial and posterior shaft. The most stressed configurations were the 10° anterior and 10° posterior tilts, with stress concentrated along the shaft and extending distally in both anterior and posterior views. Stresses experienced in the 10° anterior loading scenario extended further proximally than in the 10° posterior loading scenario.

#### 3.1.2 Loxodonta africana

*Loxodonta africana* exhibited the highest overall stress levels among the slim humeri. Neutral loading performed best, with 5° medial and lateral tilts showing roughly comparable performances (Figure 3). In these lower‐stress configurations, stresses were mainly concentrated along the medial shaft (Figure 4). Additional concentrations appeared at the supracondylar crest in the 10° medial, as well as the 5° and 10° lateral tests. The 5° anterior tilt exhibited predominantly anterior loading, with a uniquely isolated, anteriorly located stress concentration at the deltoid tuberosity, a pattern not observed in the other slim taxa to the same degree, alongside additional stresses along the posterior shaft. As in *L. cyclotis*, the 10° anterior and posterior tilts were the most stressed configurations, with the stresses again extending further proximally in the anterior loading scenario.

#### 3.1.3 Elephas maximus

*Elephas maximus* generally exhibited higher stress levels than *L. cyclotis*. Although the anterior view showed limited stress at a 5° anterior tilt, significant stress concentrations were present along the posterior shaft in the posterior view (Figure 4). As in *L. cyclotis*, the 10° anterior and posterior tilts were the most stressed configurations, with stress concentrated along the shaft and extending distally in both aspects. Neutral loading appeared to perform best overall, followed closely by 5° medial, lateral and anterior tilts (Figure 3). In the 5° medial tilts, stresses were primarily concentrated around the musculospiral groove and lateral epicondyle region, whereas in the 5° lateral tilt, they were distributed more along the medial shaft. During the 5° posterior tilt, stresses clustered around the distal half of the humerus on all sides.

#### 3.1.4 Mammuthus primigenius

In *M. primigenius*, neutral loading resulted in the lowest overall stress levels. The general pattern was similar to that observed in the other slim taxa, with anterior and posterior tilts producing higher stresses (Figure 3). Notably, stress was largely absent from the posterior aspect of the humerus in most configurations, except for the 5° and 10° anterior and posterior tilts, and to a lesser extent in the 10° medial and lateral tilt scenarios (Figure 4). Overall, stresses tended to be concentrated anteriorly and distally. Under lateral tilting, stresses were more pronounced on the medial side of the shaft, whereas under medial tilting, they shifted towards the lateral side. The 10° anterior and posterior tilts were the most stressed configurations.

### 3.2 Robust humeri

**FIGURE 5:**
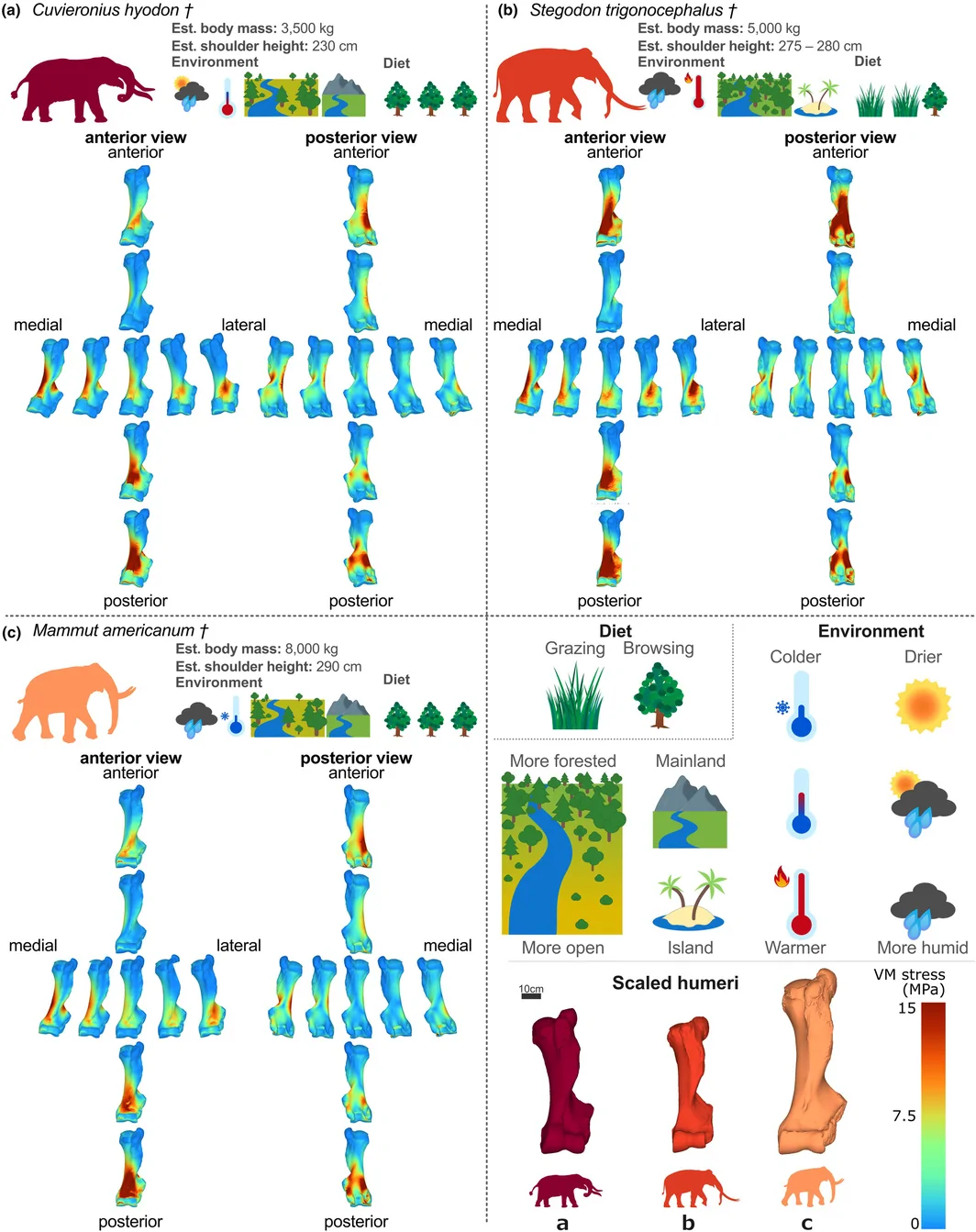
Results of the finite element (FE) analyses for the species exhibiting the robust morphotype (a, *Cuvieronius hyodon*; b, *Stegodon trigonocephalus*; c, *Mammut americanum*). Species marked with a dagger (†) are extinct. Each panel highlights the diet and environment, as well as the relative size of the humeri to each other. Estimates of body mass and shoulder height were obtained from Larramendi (2016), based on Grade I (‘average‐sized’) male specimens, when possible.

Robust humeri generally experienced lower von Mises stresses compared with slim humeri (Figure 5). Neutral loading and moderate medial and lateral tilts performed well consistently, while anterior and posterior orientations were the most stressed orientations. While the posterior orientation tended to be the most stressed in the slim morphotype, *S. trigonocephalus* exhibits the highest stresses in the 10° anterior loading (Figure 3).

#### 3.2.1 Cuvieronius hyodon

In *C. hyodon*, neutral loading and the 5° medial and lateral tilts showed similarly low stress levels and performed best overall. The 5° anterior tilt also performed well, whereas the 10° anterior tilt showed increased stress along the medial shaft (Figure 5). Among the side tilts, the 10° lateral tilt was the most stressed, with stress concentrated around both the medial and lateral shaft. The 10° posterior tilt performed worst, with stress increasing from 5° to 10°. In these configurations, stress distributions along the anterior of the shaft were similar between 5° and 10°, while stresses along the posterior shaft were limited at 5° but substantially increased at 10°.

#### 3.2.2 Stegodon trigonocephalus

The humerus of *S. trigonocephalus* was the most stressed among the robust taxa. As seen in other species, neutral loading and the 5° medial and lateral tilts performed best. The anterior view of the 5° anterior tilt showed the lowest stress levels of all configurations, although posterior views showed slightly elevated stresses along the posterior shaft (Figure 5). This pattern differs from the other robust taxa, in which stresses under anterior tilting were more concentrated along the medial shaft; in *S. trigonocephalus*, stresses were instead more posteriorly distributed. *Stegodon trigonocephalus* differs from the other robust taxa by having a higher adjusted internal energy at 5° medial tilt than the others have at 10°, and its performance at 10° medial tilt is poor enough to cluster with the slim morphotype at 5° (Figure 3).

#### 3.2.3 Mammut americanum

The overall stress patterns in *M. americanum* closely resembled those of *C. hyodon*. Stress magnitudes were generally slightly lower (compared with *C. hyodon*) across all configurations. As in the other robust taxa, neutral loading and moderate (5°) medial, lateral and anterior tilts performed best, while posterior tilts were the most stressed orientations. For the 10° anterior tilts, peak stresses concentrated along the distal medial/posterior shaft, and to a lesser degree on the transition between the anterior shaft of the humerus and the medial aspect of the radial fossa. 10° posterior loading resulted in similar but more intense loading patterns, in addition to stress concentrations surrounding the olecranon fossa (Figure 5).

## 4 DISCUSSION

Slender and robust proboscidean humeral morphotypes exhibit distinct stress distributions under axial compression. Deviating from the neutral alignment significantly impacts the mechanical response, with the robust morphotype consistently outperforming slender morphologies. In addition, clear morphological and associated biomechanical responses are present within morphotypes, suggesting function‐driven interspecific variation among proboscideans.

### 4.1 Stance, alignment and columnar limb function

Across the seven proboscidean species considered in this study, von Mises stress magnitudes generally increased as the humerus deviated from a neutral (0°) orientation to a tilted loading scenario, reinforcing the functional importance of maintaining near‐columnar limb alignment in large quadrupeds. Columnar posture reduces bending moments by aligning joint reaction forces (JRFs) more closely with the long axis of the bone (Christiansen, 2007; Kokshenev & Christiansen, 2010), thereby minimising tensile and compressive asymmetries across the cortex. Previous work on extant proboscidean limb compliance has shown that forelimb elements are stiffer than hindlimb elements, presumably in response to the more cranially positioned centre of mass in elephants, thereby making them particularly adapted to resist increased compressive loads (Kokshenev & Christiansen, 2010). Our simulations highlight the importance of limb alignment under compressive loading conditions, showing that even modest deviations from axial loading substantially increase stress concentrations and underscoring the mechanical consequences of departing from a columnar stance (Figure 3; Figure S2).

An increased diaphyseal cross‐sectional area is presumed to provide a mechanical buffer against mediolateral and anteroposterior bending stresses. Accordingly, compressive loading scenarios for the robust morphotype consistently distributed stresses more evenly and tolerated off‐axis loading better than the slender morphotype. In large mammals, such enhanced resistance may be particularly important in dynamic conditions, such as turning, substrate irregularities or muscular stabilisation during locomotion.

### 4.2 Biomechanical trade‐offs and ecological implications

Bone adapts to its mechanical environment through modelling and remodelling processes in response to straining (Robling et al., 2006; Squire et al., 2008; Sugiyama et al., 2010). In general, bone is deposited in regions experiencing high stresses and strains (Sugiyama et al., 2010), and resorbed where strain is low (Squire et al., 2008); a mechanism maintaining stresses within a safe range while minimising unnecessary metabolic costs associated with excess bone tissue (Doube et al., 2011; Robling et al., 2006). The distinction between robust and slender humeri highlights a fundamental structural trade‐off. Increased robusticity improves resistance to axial compression and bending by reducing peak stress under equivalent loading. However, greater bone mass increases inertial resistance and the energetic expense of limb oscillation and maintenance (Campione & Evans, 2012; Myers & Steudel, 1985; Schmidt‐Nielsen, 1984; Steudel, 1990). Comparative analyses across mammals demonstrate that variation in limb bone slenderness reflects a balance between mechanical strength and locomotor efficiency (Martín‐Serra et al., 2014). Slender bones may reduce distal limb mass and improve energetic efficiency, whereas robust bones provide higher safety factors against loading extremes. In proboscideans, this trade‐off is expressed at extreme body sizes, where the risk of structural failure is higher and energetic costs are magnified.

Budd et al. (2020) note that the African savannah elephant *L. africana* and the African forest elephant *L. cyclotis* show distinct morphological differences, such as a reduced body size and downward‐facing tusks in *L. cyclotis*, enabling easier passage through its thickly forested habitat (Budd et al., 2020; Roocroft & Oosterhuis, 2000). A reduced body size compared with *L. africana* is also observed in the Asian elephant *E. maximus* (Bader et al., 2023; Larramendi, 2016), which primarily inhabits humid tropical forests characterised by soft, compliant and uneven substrates, whereas *L. africana* occupies more open environments, including savanna grasslands, sandy plains and hard, arid surfaces (Bader et al., 2025; Roocroft & Oosterhuis, 2000). The superior performance of *L. cyclotis* and *E. maximus* in off‐axis orientations compared with *L. africana* might reflect an additional functional adaptation in the limb bones, resulting in increased mobility in traversing dense arboreal environments (Figure 3). Locomotion in forested habitats likely requires increased limb stabilisation and fine motor control to navigate irregular ground and dense vegetation (Bader et al., 2023, 2025). In addition to these environmental differences, the species exhibit distinct foraging behaviours. Asian elephants frequently use their forefeet to scrape and excavate soil, performing forceful, repetitive forelimb movements. In contrast, African savanna elephants are primarily browsers and rely predominantly on their trunks rather than their feet during foraging (Clemens & Maloiy, 1982; Roocroft & Oosterhuis, 2000). The broader range of forelimb motion associated with substrate manipulation in *E. maximus* may increase muscular loading, potentially contributing to the comparatively robust humeral morphology observed in this species (Bader et al., 2023, 2025). *Loxodonta cyclotis* and *E. maximus* also show comparable performance in off‐axis orientations (Figure 4), suggesting that similar functional demands may underlie shared mechanical response.

Despite their disadvantages under compressive loads, the slender build of woolly mammoths likely enhanced locomotor efficiency. Longer, more gracile limbs facilitate longer strides, enabling these animals to traverse vast Pleistocene steppe environments in search of sparse resources (Belyaev et al., 2024). This increase in locomotor efficiency would have been advantageous for seasonal migrations (Hodgson et al., 2008; Wooller et al., 2021). Alongside the woolly mammoth (*M. primigenius*), the mammoth lineage includes various species with different body proportions, ranging from the insular dwarf mammoths (*Mammuthus lamarmorai*, *Mammuthus creticus* and *Mammuthus exilis*) to the large southern mammoth (*Mammuthus meridionalis*) and steppe mammoth (*Mammuthus trogontherii*), each occupying different ecological niches. The North American mammutids (genus *Mammut*, Mammutidae), not to be confused with mammoths (*Mammuthus*, Elephantidae), likely browsed in dense coniferous forests (Birks et al., 2018; Green et al., 2005; Hodgson et al., 2008; Newsom & Mihlbachler, 2006) and therefore may not have benefited from greater height and stride lengths. A wide pelvis and robust limb structure (Larramendi, 2016) could have facilitated improved movement over uneven forest terrain, in contrast to woolly mammoths' slimmer, energy‐efficient builds adapted for open landscapes. Greater shoulder height raises the centre of mass, which reduces stability and increases both the likelihood and potential severity of falls, particularly in large‐bodied organisms (Hutchinson, 2021). As woolly mammoths retained a slender build, this likely reflects a trade‐off favouring energy‐efficient locomotion over structural robustness. In contrast, mammutids and similarly robust taxa benefited from a skeletal morphology that provided greater structural strength and a lower centre of mass.

A different ecological context applies to *S. trigonocephalus* from Java, Indonesia. Although Java is an island today, this landmass was connected to the mainland at various points during the Pleistocene, resulting in a habitat less isolated than that of other Indonesian islands, such as Flores. Dwarfing in *S. trigonocephalus* is also not as pronounced as in *Stegodon florensis* and *Stegodon sondaari* (Larramendi, 2016), although *S. trigonocephalus* still shows a distinct reduction in body size. As a species restricted to Java, its morphology may have been influenced by island‐specific evolutionary pressures, including processes related to the ‘island rule’ (Herridge, 2010; Lomolino et al., 2006), which can alter body size and skeletal proportions in ways not directly linked to functional performance. Various subspecies have been proposed for *S. trigonocephalus* on Java (van den Bergh, 1999), indicating that this species' habitat was insular at various stages during the Early Pleistocene (Puspaningrum et al., 2020), although not as isolated as that of *S. floresiensis* or some of the endemic proboscidean fauna on Mediterranean islands. Altering life‐history traits have been proposed for the dwarfed proboscideans, including the Sicilian dwarf *Palaeoloxodon falconeri*, which shows a remarkably slow growth rate and clear lines of arrested growth within the long bones (Köhler et al., 2021). As a proposed insular species, the lack of migratory opportunities, alongside typical insular constraints (e.g. resource limitation; Herridge, 2010; Lomolino, 2005; Lomolino et al., 2006; McNab, 2002), might affect the functional development of the limb bones. Interpreting the functional significance of skeletal robustness in *S. trigonocephalus*, therefore, remains complicated by its insular context. *Stegodon trigonocephalus* inhabited an environment undergoing a transition from dense forests to more open savanna–woodland systems (Amiruddin et al., 2025; Puspaningrum et al., 2020). Isotopic evidence suggests that *S. trigonocephalus* was primarily a specialised grazer adapted to a diet based on C4 plants (Puspaningrum et al., 2020). Within these environments, it likely faced substantial ecological competition from other large herbivores, including deer, large bovids, elephants (*Elephas hysudrindicus*) and rhinoceroses (*Rhinoceros sondaicus*), as well as potential predation pressures from large carnivores, such as tigers and possibly *Homo erectus* (Puspaningrum et al., 2020; van der Geer et al., 2016). The coexistence of *S. trigonocephalus* with *E. hysudrindicus* suggests that these taxa may have occupied overlapping grazing niches, potentially intensifying resource competition, particularly given that *E. hysudrindicus* was likely a more generalist herbivore (Puspaningrum et al., 2020). Alongside size variation, the humerus of *S. trigonocephalus* appears comparatively robust, whereas other limb elements analysed by Bader et al. (2024) show more intermediate proportions. This suggests that postcranial robustness in this taxon may have been unevenly distributed across the skeleton, obscuring ecological interpretations. The intermediate nature of the *S. trigonocephalus* humerus is further supported by the adjusted internal energy values derived from the FEA (Figure 3). Although it is classified as a robust humerus by Bader et al. (2024), its performance at 5° medial tilt is notably poorer than that of the other robust specimens at 10° medial tilt. This discrepancy suggests that it does not fully conform to the functional expectations of robust morphologies. Instead, it may represent a true intermediate structural strategy, potentially reflecting adaptive pressures associated with its insular habitat. Together, these factors indicate that the functional and ecological drivers of skeletal morphology in *S. trigonocephalus* may differ from those observed in mammoths and mammutids inhabiting continental environments.

### 4.3 Multidirectional loading and impact of bone microstructure

The improved performance of robust humeri under mediolateral and anteroposterior tilts suggests adaptation to multidirectional bending stresses. Increased cortical thickness and expanded cross‐sectional geometry may therefore reflect adaptation to gravitational (and muscular) stresses.

Microanatomical analyses of extant elephant long bones reveal thick cortical regions and trabecular architectures aligned with principal loading directions, indicating structural adaptation to habitual stress regimes (Bader et al., 2025). Both *L. africana* and *E. maximus* exhibit greater cortical thickness on the medial side of the humerus and at the deltoid tuberosity, as well as a trabecular structure oriented from the thicker medial cortex to the more distal, lateral epicondylar crest (Bader et al., 2025). This provides greater resistance to axial compression (Bader et al., 2025). Our FEA results complement these findings by showing how the orientation of external loading interacts with external morphology to shape stress patterns. The main stress concentrations were located on the medial shaft and near the deltoid tuberosity (Figure 4), corresponding to areas of increased cortical thickness observed by Bader et al. (2025).

Additionally, the stress on the lateral supracondylar crest (Figure 4) is evidently compensated for, to some degree, by the trabecular structure oriented in the direction of loading (as seen in Bader et al., 2025). This suggests that humeral microstructure may compensate for mechanically vulnerable regions created by external morphology, reinforcing areas of elevated stress and thereby reducing failure risk. Robust morphotypes appear to maintain lower peak stresses under diverse loading conditions, potentially reflecting higher overall safety factors. Whether species with the robust morphotype also exhibit this increased cortical bone thickness, or instead compensate for a thinner cortex by developing more robust humeri, remains unclear. Integration of microanatomical structures within both extant and extinct proboscideans would further aid interpretations of humeral biomechanical performance.

Bone compliance estimations in *L. africana* and *E. maximus* predicted that *E. maximus* had more compliant limb bones than *L. africana*, with the increase in stiffness in *L. africana* increasing resistance to axial compression (Bader et al., 2023; Kokshenev & Christiansen, 2010). Our results indicate a similar pattern: *L. africana* performs better in a neutral non‐tilted orientation than *E. maximus*, whereas *E. maximus* experiences comparatively less stress when deviating from the neutral alignment (notably the anterior, medial and lateral orientations; Figure 4).

### 4.4 Methodological implications and broader applications

Beyond its biological implications, this study establishes a replicable framework, especially for simulating compressive loading in columnar‐limbed, graviportal taxa. By systematically varying humeral orientation under axial compression, the approach evaluates bone performance across a realistic range of postural variation rather than relying on a single idealised configuration. Because limb orientation inevitably varies during locomotion and weight support, modelling a range of orientations provides a more robust assessment of structural performance under functional loading conditions.

The loading setup used here is biomechanically appropriate for other graviportal animals. Species with predominantly columnar limb postures experience loading regimes during stance that are largely dominated by axial compression (Christiansen, 2007; Kokshenev & Christiansen, 2010). We modelled the humerus in a vertical orientation, reflecting the most common loading condition in elephants, given their graviportal limbs. Under these conditions, compressive loading provides a reasonable approximation of the mechanical environment the limb experiences during weight support. Stress patterns predicted by the models correspond closely with independently observed patterns of structural reinforcement within the bone. Regions experiencing elevated stress align with areas of increased cortical thickness identified through microanatomical analyses (Bader et al., 2025). This form–function relationship provides independent validation of the modelling framework. If the applied loading conditions did not approximate realistic biomechanical conditions, predicted stress concentrations would not be expected to coincide with anatomical reinforcement. The co‐occurrence of the simulated stress distributions and observed bone structure strongly suggests that the finite element models presented herein capture key aspects of the humerus' mechanical environment.

Our results demonstrate that applying compressive loading to limb bones of graviportal animals, while systematically varying limb orientation, provides a rigorous approach to assessing the implications of long bone morphology for biomechanical performance. The framework is readily replicable and offers a standardised method for evaluating compressive performance in large‐bodied taxa, enabling meaningful comparisons between skeletal elements or across species with similar locomotor strategies.

Because the modelling approach focuses on compressive loading in columnar limbs, it is readily transferable to other large‐bodied taxa that exhibit similar postural mechanics. In addition to other limb elements within proboscideans, the framework can be extended to comparable graviportal lineages, such as sauropod dinosaurs, which independently evolved columnar limb postures to support extreme body mass (Marcé‐Nogué, 2022). Although quantitative biomechanical analyses of compressive loading in heavy‐bodied taxa remain relatively limited, recent work has begun to apply similar loading frameworks to questions of locomotion and behaviour in sauropods (Silva Junior et al., 2025). More broadly, the method proposed here provides a consistent basis for examining how heavy terrestrial animals accommodate extreme loads through skeletal structure, facilitating comparative analyses across lineages with similar functional constraints.

As with all biomechanical modelling approaches, certain assumptions constrain interpretation. Material properties are simplified, and the models do not account for various parameters, such as muscle architecture, JRF or bone microstructure. The absence of JRFs and muscular loads is expected to change stress concentrations (Etienne et al., 2024, 2025). However, due to the lack of associated specimens and detailed soft‐tissue information, incorporating these factors would make the study more speculative. Other aspects, such as intraspecific variation related to sexual dimorphism or geographic variability, were not taken into account as our dataset comprised only one specimen per species. Instead, this work serves as a proof of concept for future case studies where a large‐scale study of extant elephantids can assess functional variation within proboscidean taxa and define distinct variation in performances between and within species. In addition, the method is most appropriate for taxa that maintain relatively columnar limb postures, as species with highly flexed or crouched limb mechanics would require different boundary conditions and loading regimes. Nevertheless, the strong correspondence between simulated stress distributions and anatomical reinforcement demonstrates that this approach provides a reliable and informative framework for investigating limb bone mechanics in graviportal taxa.

## 5 CONCLUSION

Slender and robust morphotypes of the humerus in proboscideans respond differently to compressive loading. This study confirms that maintaining a near‐columnar limb orientation is critical for minimising stress, reinforcing the importance of posture in supporting extreme body mass. Robust humeri exhibit superior performance under both axial and off‐axis loading, indicating greater tolerance to multidirectional stresses. In contrast, slender morphologies appear more optimised for efficient locomotion under near‐axial loading, at the cost of reduced structural resilience when alignment deviates from the neutral alignment.

These findings highlight a fundamental biomechanical trade‐off between strength and robustness versus the efficiency of maintenance and locomotion. Variation in humeral performance across taxa suggests that ecological factors, such as habitat complexity and substrate variability, play a significant role in shaping these structural differences. The intermediate characteristics observed in *S. trigonocephalus* emphasise that these morphotypes represent a spectrum of adaptive strategies, rather than discrete categories.

Methodologically, this study establishes a robust and transferable framework for analysing compressive loading in columnar‐limbed taxa. By incorporating variation in limb orientation, the approach captures a more realistic range of functional conditions and can be extended to other graviportal lineages, including extinct megafauna. Despite inherent simplifications, the strong correspondence between predicted stress patterns and known anatomical reinforcement suggests that the model outputs are anatomically coherent. This work advances our understanding of how proboscideans accommodate extreme mechanical demands through skeletal modification, offering broader implications for the study of locomotion and functional morphology in large, terrestrial vertebrates.

## AUTHOR CONTRIBUTIONS

JJH provided supervision, resources and project administration. NC provided the co‐supervision. NFW and JJH scanned the models. NFW and NC prepared them for finite element analysis. NFW, NC and JJH developed the methodological framework. NFW carried out the finite element analyses and wrote the initial draft. All authors designed the initial study and overarching research questions, reviewed and revised the original draft, and gave their final approval for the submitted version.

## FUNDING INFORMATION

This work was supported by the Belgian American Educational Foundation (BAEF), the National Science Foundation (NSF DBI‐2128146) and European Union HORIZON‐MSCA‐2024‐PF‐01 (grant agreement no.: 101207039).

## Supporting information


**Data S1.** ESM Metafor_ElephasMaximusInputFile.

**Table S1.** Internal energy (in mJ) of each species for each loading orientation, as well as the volume of the model after scaling to the same length (766 mm).
**Table S2.** Adjusted internal energy (mJ/mm^3^) of each species for each loading orientation, as well as the volume of the model after scaling to the same length (766 mm).

**Figure S1.** Annotated humerus of *Elephas maximus* from the anterior (left) and posterior (right) views.
**Figure S2.** Results of the FE analyses for the slim (A, *L. africana*; B, *L. cyclotis*; C, *E. maximus*; D, *M. primigenius*) and robust (E, *C. hyodon*; F, *S. trigonocephalus*; G, *M. americanum*) morphotypes. Panel (H) highlights the geographic and temporal distribution, diet and environment of the studied species, as well as the relative size of the humeri to each other.
**Figure S3.** Displacement (in mm) in the *Z*‐axis direction for each species, derived from the FE analyses.

## Data Availability

The 3D models used in this study are available on www.morphosource.org. Specimens scanned for the purpose of this study (*Stegodon trigonocephalus*, *Mammuthus primigenius*, *Mammut americanum* and *Loxodonta cyclotis*) are uploaded into the Proboscidean Humeri Project (ID: 000853405). Models scanned by other researchers are also available on the MorphoSource repository: the *Elephas maximus* humerus was provided by the Idaho Museum of Natural History (funded by the Rick Carron Foundation: NSF DBI‐1802491) (ID: 000168768), *Loxodonta africana* (mnhn:zm:AC‐1907‐49; MNHN—Paris) (ID: 000632460) and *Cuvieronius hyodon* (mad:f:TAR2514; MNHN—Paris) (ID: 000631088). The finite element models used in this study are available at the institutional repository of the University of Liège ORBI: https://orbi.uliege.be/handle/2268/348021.
