# Supplementary material for: Modelling compressive loading in large‐bodied mammals reveals distinct biomechanical strategies in proboscidean humeri
Source: J Anat. 2026 Sep 7:10.1111/joa.70234. Online ahead of print. doi: 10.1111/joa.70234 (PMC13550740; doi:10.1111/joa.70234)
Supplement: Supplementary file 1 [file JOA-9999-0-s001.pdf]

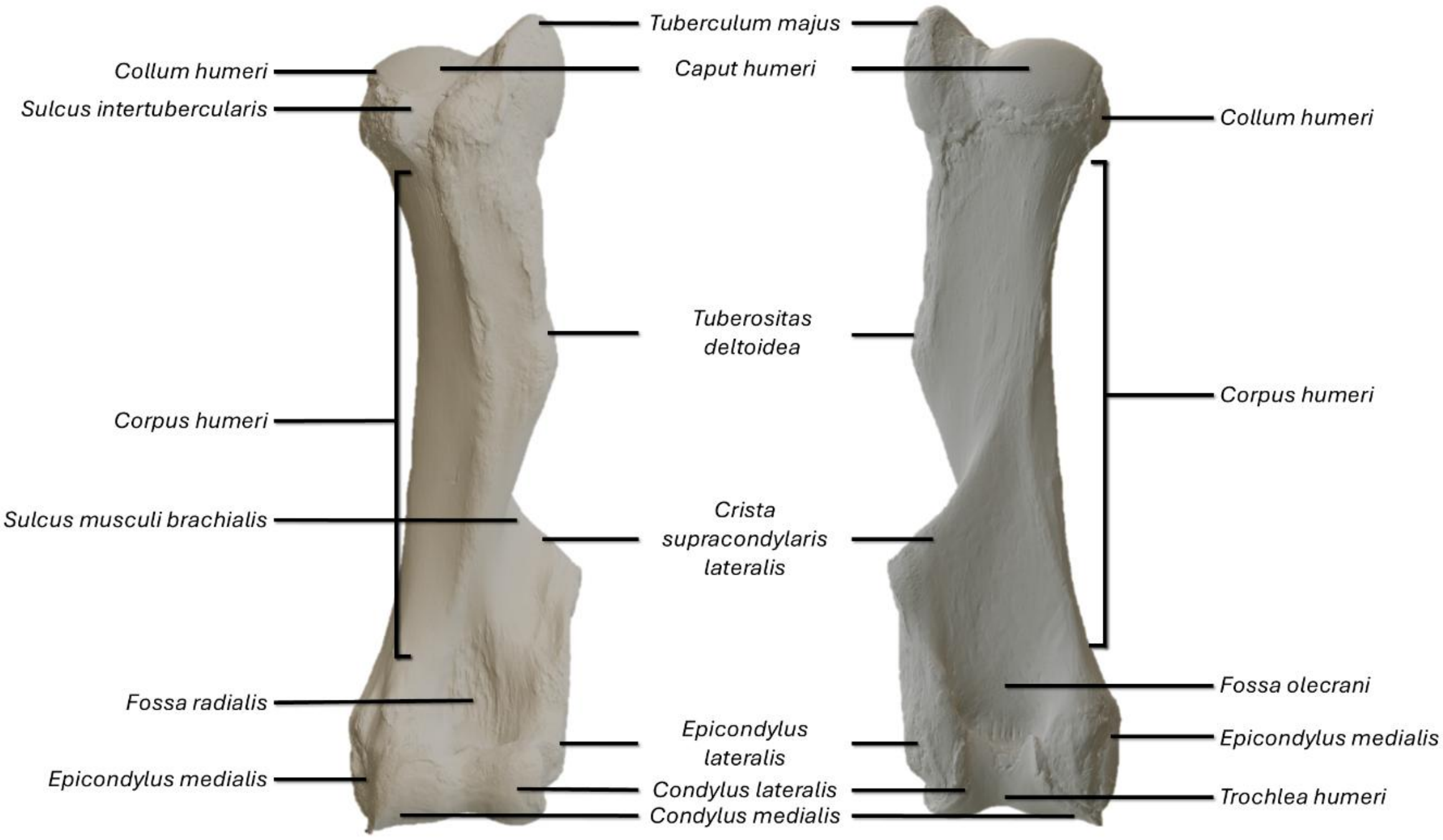

Figure S1. Annotated humerus of *Elephas maximus* from the anterior (left) and posterior (right) views.

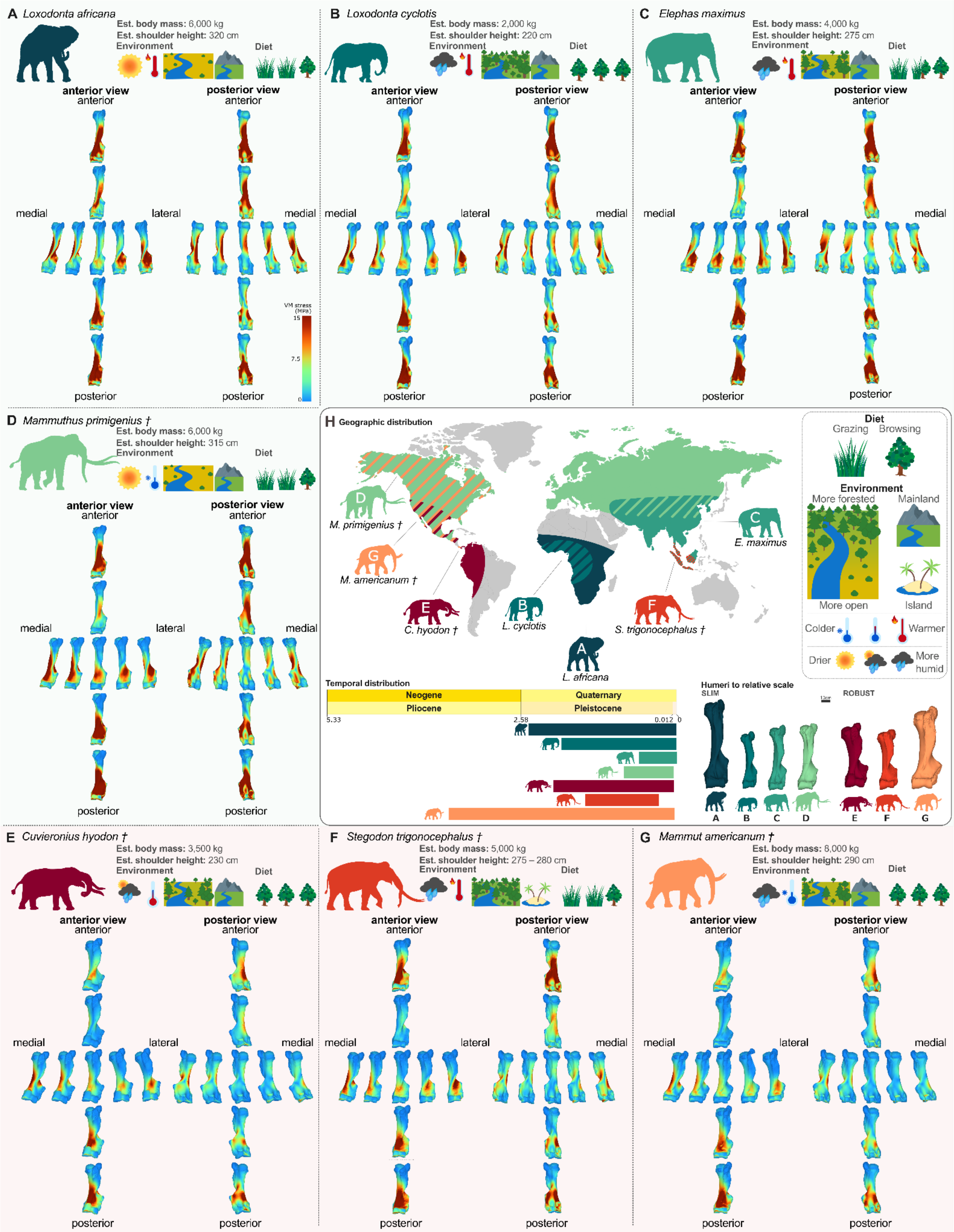

Figure S2. Results of the FE analyses for the slim (**A.** *L. africana*; **B.** *L. cyclotis*; **C.** *E. maximus*; **D.** *M. primigenius*) and robust (**E.** *C. hyodon*; **F.** *S. trigonocephalus*; **G.** *M. americanum*) morphotypes. Panel **H.** highlights the geographic and temporal distribution, diet, and environment of the studied species, as well as the relative size of the humeri to each other. Estimates of body mass and shoulder height were obtained from Larramendi (2016), based on Grade I ('average-sized') male specimens, when possible.

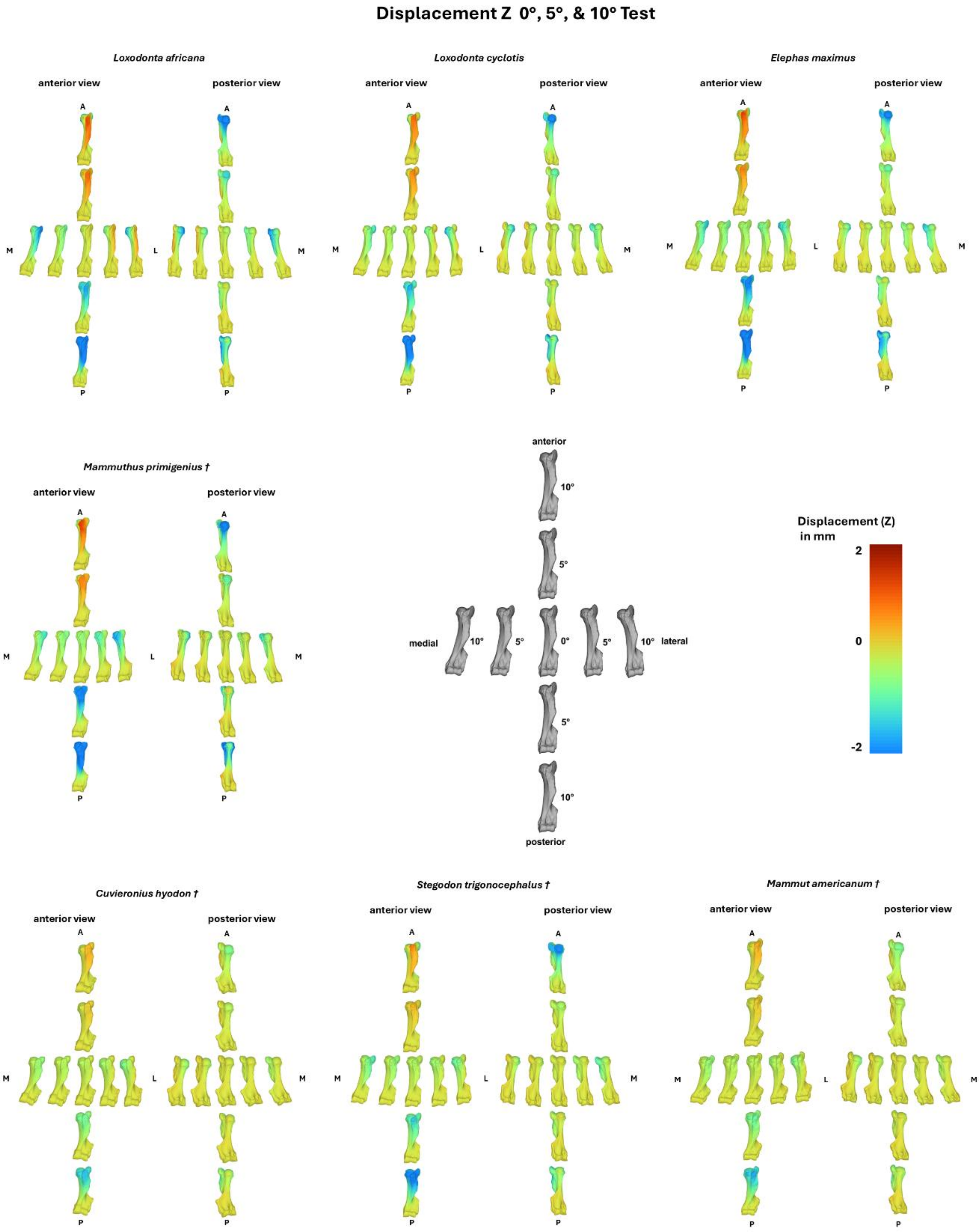

Figure S3. Displacement (in mm) in the Z-axis direction for each species, derived from the FE analyses. For each species, the anterior view (left) and posterior view (right) are shown, with 0°, 5°, and 10° tilts in each direction. A greyscale image illustrates the tilting regime in anterior view.
